# Interferometric Study of Ionospheric Plasma Irregularities in Regions of Phase Scintillations and HF Backscatter

**DOI:** 10.1029/2021GL097013

**Published:** 2022-06-16

**Authors:** Andres Spicher, James LaBelle, John W. Bonnell, Roger Roglans, Chrystal Moser, Stephen A. Fuselier, Scott Bounds, Lasse B. N. Clausen, Francesca Di Mare, Connor A. Feltman, Yaqi Jin, Craig Kletzing, Wojciech J. Miloch, Jøran I. Moen, Kjellmar Oksavik, Rhyan Sawyer, Toru Takahashi, Tim K. Yeoman

**Affiliations:** ^1^ Department of Physics and Technology UIT the Arctic University of Norway Tromsø Norway; ^2^ Department of Physics and Astronomy Dartmouth College Hanover NH USA; ^3^ Space Sciences Laboratory University of California Berkeley Berkeley CA USA; ^4^ Southwest Research Institute San Antonio TX USA; ^5^ Department of Physics and Astronomy University of Texas at San Antonio San Antonio TX USA; ^6^ University of Iowa Iowa City IA USA; ^7^ Department of Physics University of Oslo Oslo Norway; ^8^ University Centre in Svalbard Longyearbyen Norway; ^9^ Birkeland Centre for Space Science Department of Physics and Technology University of Bergen Bergen Norway; ^10^ Electronic Navigation Research Institute National Institute of Maritime, Port, and Aviation Technology Tokyo Japan; ^11^ Physics and Astronomy University of Leicester Leicester UK

## Abstract

We investigate the nature of small‐scale irregularities observed in the cusp by the Twin Rockets to Investigate Cusp Electrodynamics‐2 (TRICE‐2) in regions of enhanced phase scintillations and high‐frequency coherent radar backscatter. We take advantage of the fact that the irregularities were detected by spatially separated probes, and present an interferometric analysis of both the observed electron density and electric field fluctuations. We provide evidence that fluctuations spanning a few decameters to about a meter have low phase velocity in the plasma reference frame and are nondispersive, confirming that decameter‐scale irregularities follow the **E** × **B** velocity. Furthermore, we show that these “spatial” structures are intermittent and prominent outside of regions with strongest precipitation. The observations are then discussed in the context of possible mechanisms for irregularity creation.

## Key Points

- The phase velocity and wavelength of coexisting ionospheric cusp density and electric field fluctuations is determined using interferometry
- In‐situ confirmation of decameter‐–meter‐scale density and electric field fluctuations having low phase velocity in the plasma frame
- Intermittent decameter‐scale fluctuations coincide with larger‐scale density variations outside of enhanced precipitation regions

## 1 Introduction

Turbulent flows generally exhibit spatio‐temporal randomness or an “irregularity” character spanning a wide range of scales (Tennekes et al., 1972; Tsinober, 2009). In the ionosphere, such irregular structures are common (Kelley, 2009), and meso‐ to small‐scale fluctuations in electron density (*N*_*e*_) are of particular interest for space weather. Indeed, *N*_*e*_ fluctuations of the order of hundreds of meters to a few kilometers can affect Global Navigation Satellite Systems signals and cause “scintillations” (rapid fluctuations in signal amplitude or phase) (Hey et al., 1946; Kintner et al., 2007; Yeh & Liu, 1982). Furthermore, decameter‐scale structures cause high frequency (HF) backscatter detected by the Super Dual Auroral Radar Network (SuperDARN) (Greenwald et al., 1995). With increasing need for reliable Arctic communication and navigation systems, a detailed understanding of these irregular structures and their cause(s) is essential (e.g., Moen et al., 2013).

Of particular interest are the ionospheric cusp regions, where irregularities and phase scintillations peak (Heppner et al., 1993; Jin et al., 2015, 2019), and where SuperDARN echoes have wide and complex Doppler spectra (Baker et al., 1995; Moen et al., 2002; Villain et al., 2002). Processes such as the gradient‐drift instability (GDI) (Tsunoda, 1988), particle precipitation (Dyson & Winningham, 1974; Moen et al., 2002; Ponomarenko et al., 2007), and inhomogeneous flows (Basu et al., 1994; Heppner et al., 1993; Spicher et al., 2020) are considered important. However, the dominant mechanism(s) are not yet assessed (e.g., Chisham et al., 2007; Moen et al., 2013; Ponomarenko & Waters, 2006).

To advance our understanding, it is essential to characterize properties of the fluctuations involved (e.g., Kintner & Seyler, 1985; LaBelle & Kintner, 1989). In this study, we investigate the nature of irregularities detected in the cusp by TRICE‐2. While the interpretation of turbulence observations by single probes on moving spacecraft requires a priori assumptions about the fluctuations (Fredricks & Coroniti, 1976; Temerin, 1978), we take advantage of the fact that irregularities were detected by spatially separated probes. We present interferometric analysis of co‐existing *N*_*e*_ and electric field (*E*‐field) fluctuations observed within regions of enhanced phase scintillations and wide SuperDARN spectra, and estimate their phase velocities and corresponding wavelength. Our analysis shows the first in‐situ experimental evidence of such decameter‐scale irregularities (reaching scales shorter than the oxygen gyroradius) being “frozen in” in the cusp *F* region, an assumption essential for SuperDARN convection maps. The observations are then discussed in the context of possible mechanisms for irregularity creation.

## 2 Instrumentation

TRICE‐2 was part of the Grand Challenge Initiative Cusp and consisted of two sounding rockets launched from Andøya, Norway, on 08 December 2018: a high‐flyer (T2‐H) reaching an apogee of ∼1,042 km, and a low‐flyer (T2‐L) reaching ∼757 km altitude. We focus here on the multi‐needle Langmuir probe (mNLP) system (Bekkeng et al., 2010; Jacobsen et al., 2010) and the *E*‐field instrument. For more information about TRICE‐2, see Moser et al. (2021) and Sawyer et al. (2021).

The mNLP system consisted of four cylindrical Langmuir probes with diameter of 0.51 mm and length of 39 mm. Fixed bias voltages (3, 4.5, 6, 7.5 V) were applied to the probes, allowing *N*_*e*_ determination at a cadence of 10 kHz (Clausen, 2022). The interferometry analysis presented below relies on probes mNLP2 (4.5 V) and mNLP3 (6 V) located on opposite sides of the payload and separated by *d* = 1 m. Results using other probe pairs generally agree. For *N*_*e*_ calculations, the spin frequency and two harmonics were removed using band‐pass filters (Jacobsen et al., 2010), and it was assumed that the probes did not act as infinitely long cylinders (assumptions: *β* = 0.8 and temperature *T_e_* = 3500 K, see Hoang et al. (2018); Marholm and Marchand (2020)).

The DC *E*‐field instrument consisted of four spherical probes mounted *d* = 6.5 m apart on booms deployed perpendicular to the payload axis. With this configuration the probe to probe *E*‐field and the probe to payload potential (ΔΦ) were determined at a cadence of 2.5 kHz.

Data from ground‐based instruments provide geophysical context. We show the 630 nm auroral emission obtained from an all‐sky imager located in Ny‐Ålesund, Norway, as well as phase scintillations indices (Fremouw et al., 1978) calculated using 1 s raw carrier phase data obtained from four receivers on Svalbard (Oksavik, 2020a, 2020b). The 1 s index (*σ*_*ϕ*1*s*_) is calculated using a sixth‐order Butterworth high‐pass filter with conventional 0.1 Hz cut‐off frequency (Van Dierendonck et al., 1993). To minimize errors, data with elevation angle >25° are used (Jin et al., 2015). We also show spectral width data from the SuperDARN (Chisham et al., 2007; Greenwald et al., 1995) radar at Hankasalmi, Finland. These data use the range‐finding algorithm with corrections for 1.5 hop ionospheric backscatter (Yeoman et al., 2008, 2012).

## 3 Background Observations

**Figure 1:**
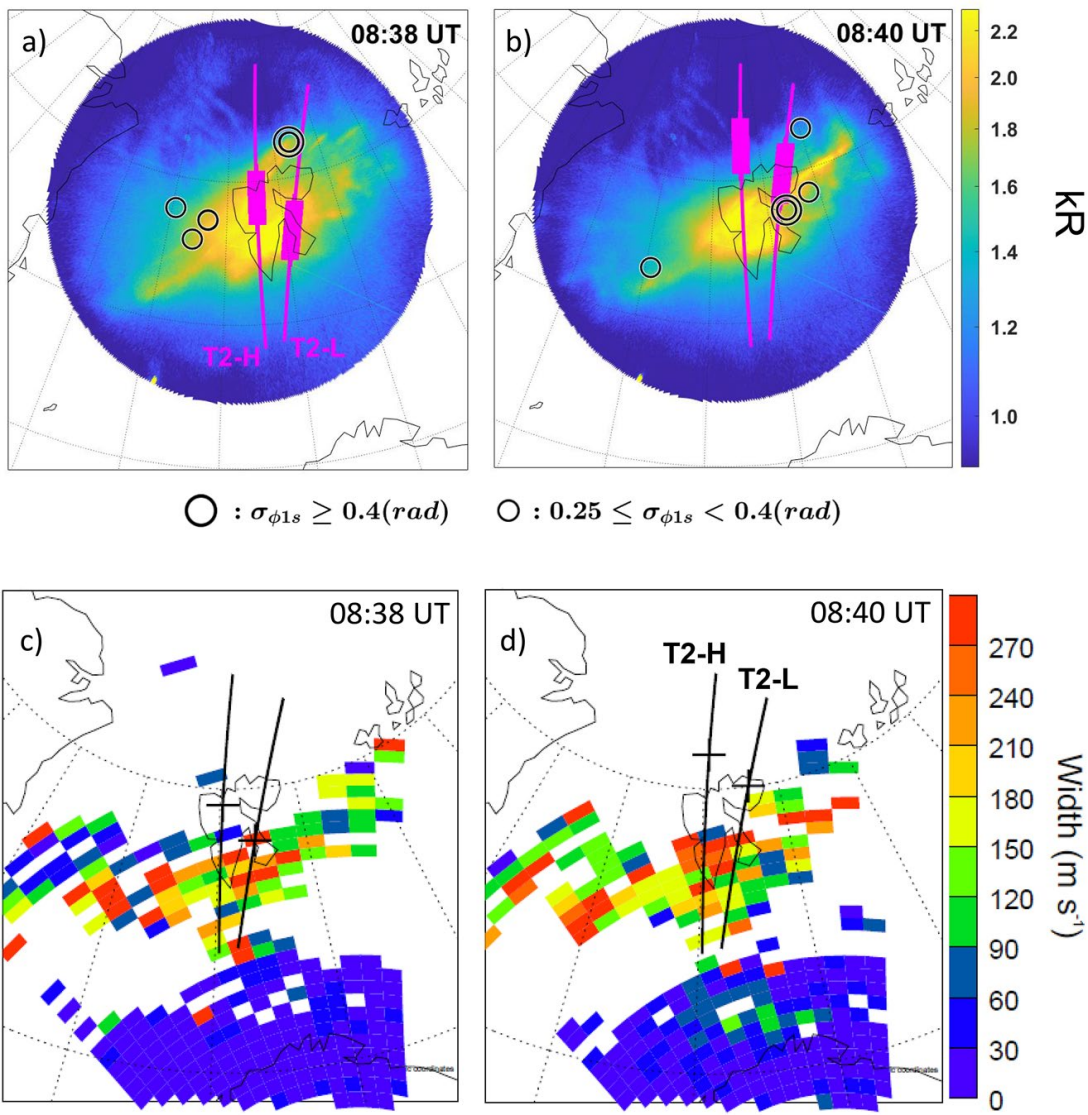
(a and b) 630 nm All‐Sky imager data projected to 250 km altitude at *t* = 08:38 UT and *t* = 08:40 UT (color‐coded) and enhanced 1 s phase scintillation indices (black circles) during *t* ± 1 min. Payload trajectories (T2‐H: high‐flyer, T2‐L: low‐flyer) are shown in magenta, with thicker lines for *t* ± 1 min. (c and d) Super Dual Auroral Radar Network spectral width with trajectories (black lines) and payload location (black crosses).

Context is provided in Figure 1. Figures 1a and 1b show 630 nm emissions at *t* = 08:38 UT and *t* = 08:40 UT, as well as *σ*_*ϕ*1*s*_ ≥ 0.25 rad calculated for *t* ± 1 min. The latter is displayed as large and small circles for strong (*σ*_*ϕ*1*s*_ ≥ 0.4 rad) and medium (0.25 ≤ *σ*_*ϕ*1*s*_ < 0.4 rad) indices, respectively, with circle centers corresponding to the pierce point projected at 350 km altitude. Parts of the payload trajectories are shown using magenta lines with the thicker portions corresponding to the payload locations during *t* ± 1 min.

Both payloads were launched into active dayside aurora. Enhanced *σ*_*ϕ*1*s*_, suggesting the presence of *N*_*e*_ irregularities ranging from a few kilometers to hundreds of meters (Jin et al., 2017; Kintner et al., 2007), occurred on the edges of the strongest aurora, especially close to T2‐L. Consequently, we focus here on observations from T2‐L.

Figures 1c and 1d shows SuperDARN spectral width with the TRICE‐2 trajectories superimposed. The payloads intersected regions of large spectral width. Coherent scattering from SuperDARN is expected when irregularities have wavelength (*λ*) matching half of the radar wavelength (Ponomarenko et al., 2007; Vallières et al., 2003), which for the current observations corresponds to *λ* ≈ 15 m. Combining ground‐based observations, we expect T2‐L to observe density structures ranging from several km to *λ* ≈ 15 m.

**Figure 2:**
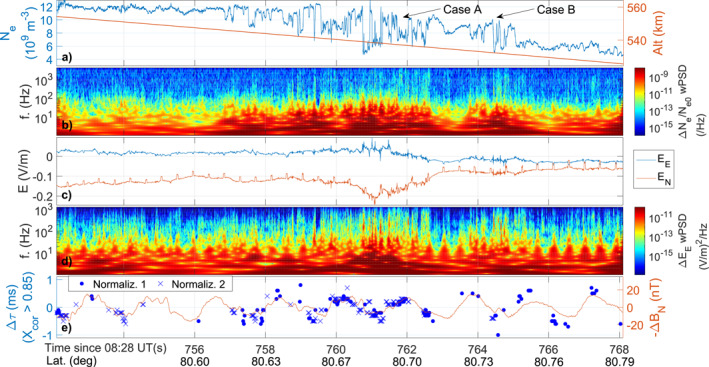
(a) *N*_*e*_ and payload altitude. (b) Wavelet spectrogram of *N*_*e*_ fluctuations. (c) Eastward (*E*_*E*_) and Northward (*E*_*N*_) components of the *E*‐field. Periodic spikes occur when an antenna probe enters the rocket's shadow. (d) Wavelet spectrogram of Δ*E*_*E*_. (e) Time shifts Δ*τ* of maximum cross‐correlation between currents fluctuations measured by two multi‐needle Langmuir probes. The red curve exhibits (median‐filtered) magnetic field fluctuations reflecting the payload spin.

Figure 2 shows data from T2‐L with respect to preliminary time of flight and latitude between 752 and 770 s (08:40:32–08:40:50 UT), that is, when T2‐L was located in the region with the large *σ*_*ϕ*1*s*_ on the poleward side of the aurora (northernmost circles in Figures 1a and 1b). Figure 2a shows *N*_*e*_ and the altitude of the payload. A large‐scale density decrease with strong fluctuations occurs along the trajectory. These fluctuations reach frequencies >1 kHz, as seen from their wavelet power spectral density (wPSD) (Torrence & Compo, 1998) in Figures 2b. Figure 2c shows the eastward (*E*_*E*_) and northward (*E*_*N*_) components of the *E*‐field. A channel of enhanced *E*_*N*_ is observed between 760 and 763 s, roughly in the center of the *N*_*e*_ fluctuations. Additionally, shorter time‐scale *E*‐field fluctuations are observed. These coincide with steep density variations/cavities and exhibit enhanced irregularity power at a few hundred Hz. This is better seen in Figure 2d showing the wPSD of Δ*E*_*E*_. Sun spikes and possibly short wavelengths attenuation effects may contribute to the periodic modulations of broadband E‐field data visible in Figure 2d.

Figures 2b and 2d show the presence of *N*_*e*_ and *E*‐field fluctuations reaching >1 kHz, with enhancements in irregularity power at a few hundred Hertz close to large *N*_*e*_‐variations. While measured in the time‐domain, in the following, we use the fact that fluctuations were measured by spatially separated probes to assess their phase velocity and corresponding wavelengths.

## 4 Interferometry Analysis of Density and Electric Field Fluctuations

### 4.1 Cross‐Correlation

The phase velocity and wavelengths of *N*_*e*_ and *E*‐field fluctuations along a rocket‐borne interferometer axis are calculated from the cross‐correlation function (*X*_*cor*_) (LaBelle et al., 1986; Pécseli et al., 1989). We computed *X*_*cor*_ for normalized current fluctuations measured by mNLP2 and mNLP3 over intervals of 0.1 s, which is much shorter than the payload spin period of about 1.78 s. Figure 2e shows the non‐zero time delays Δ*τ* of maximum *X*_*cor*_ for current fluctuations that are significantly correlated. Results using two different current normalization are shown: by removing the mean (Normaliz. 1) and a cubic spline (Normaliz. 2).

Δ*τ* follows a periodic pattern consistent with the payload spin, as seen from variations in the measured magnetic field (minus its mean value) at the spin period (Δ*B*_*N*_ in Figure 2e). This pattern means that mNLP2 observes the fluctuations before mNLP3 during half a rotation, and vice‐versa during the second half of the rotation. This sequencing is consistent with the response expected for two probes spinning in field‐aligned structures propagating from one direction perpendicular to the payload axis (LaBelle et al., 1986). From Δ*τ* and the probe separation, one can estimate the apparent velocity *v*_*ϕ*_ along the interferometer axis (LaBelle et al., 1986; Pécseli et al., 1989). For instance, Case A and B highlight features that exhibited clear small‐scale density fluctuations during different spin periods. For A, *v*_*E*×*B*_ ≈ 3,650 m/s and *v*_*ϕ*_ ≈ 3,333 m/s, while for B, *v*_*E*×*B*_ ≈ 1,900 m/s and *v*_*ϕ*_ ≈ 2,500 m/s. These features are discussed below.

### 4.2 Cross‐Spectral Density Analysis

The velocities calculated using *X*_*cor*_ are unambiguous estimates of *v*_*ϕ*_ if the coherent frequency components are phase‐shifted proportionally for each frequency (Pécseli et al., 1989). Cross‐power spectral density (CPSD) analysis in this section provides information about the phase‐shift with respect to frequencies (e.g., Kintner et al., 1984).

**Figure 3:**
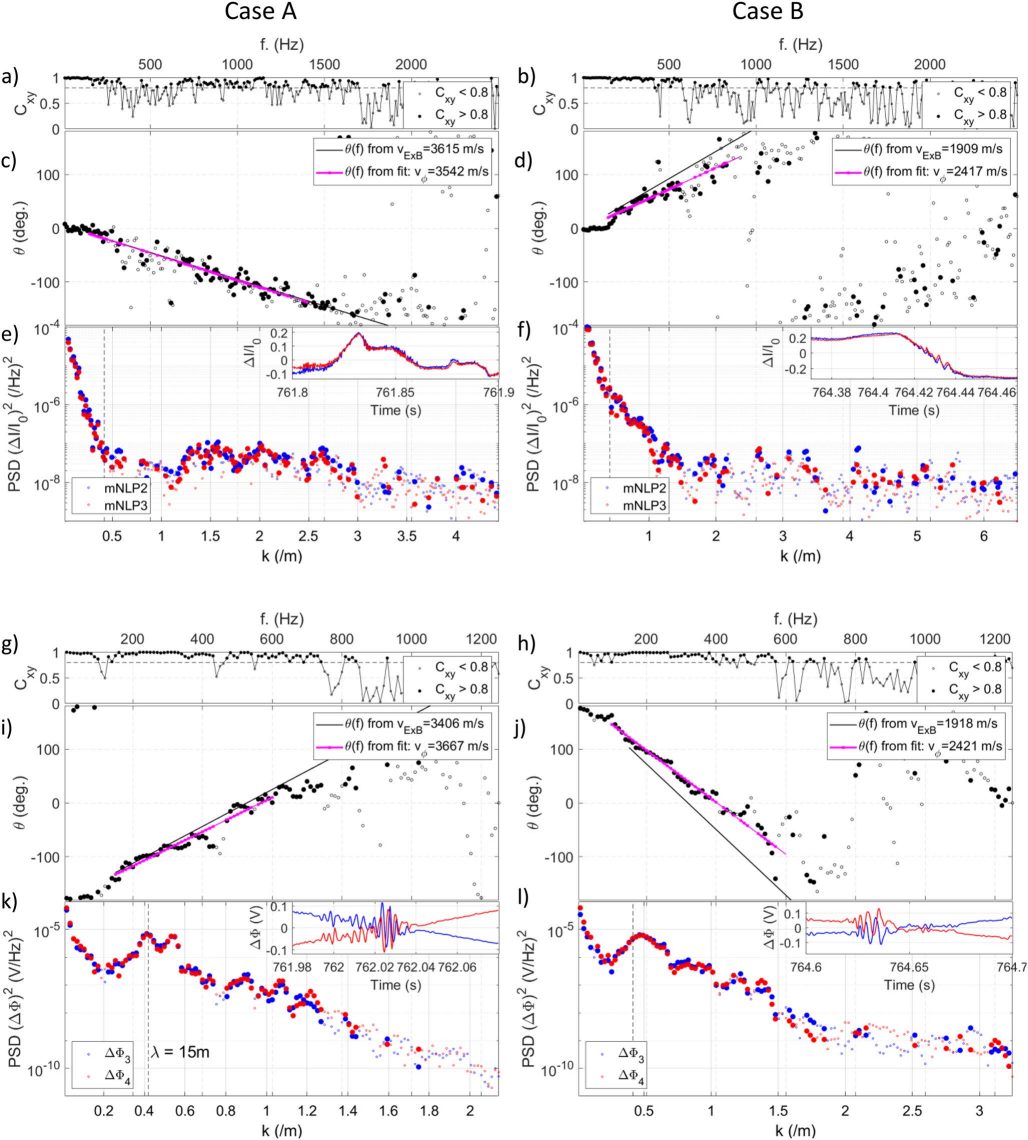
(a and b) Magnitude squared coherence (*C*_*xy*_) of relative multi‐needle Langmuir probe current fluctuations. (c and d) Relative phase angles *θ* of the current fluctuations. The black and magenta lines show *θ* obtained assuming Doppler shift due to *v*_*E*×*B*_ and from a fit, respectively. (e and f) PSD of the current fluctuations. (g–l) Similar analysis for the *E*‐field. Filled dots are used for frequency components with *C*_*xy*_ > 0.8.

Four examples are shown in Figure 3, where the left and right columns correspond to features A and B, respectively. CPSDs were computed using Matlab's build‐in function with a Tukey window with 50% overlap (Welch, 1967). Figures 3a and 3b show the magnitude‐squared coherence of the current fluctuations (shown in inlet) and Figures 3c and 3d the corresponding phase angles *θ*. Coherent frequency components are shown as filled dots. *θ* for coherent components follows a linear trend, suggesting that the structures are nondispersive (Holmgren & Kintner, 1990). The opposite sign of the slopes for each case is again consistent with Case A and B occurring in different periods of the spin.

For CPSD of *N*_*e*_ fluctuations with *λ* > *d*, the wavenumber **k** and *θ* can be related through *θ*(*ω*) = **k** ⋅ **d** (LaBelle & Kintner, 1989). Thus, for a linear dispersion relation with constant *v*_*ϕ*_ = *ω*/*k*, the velocity along the interferometer axis is given by (Bonnell et al., 1996; Holmgren & Kintner, 1990)

(1) $$v_{\phi }=2\pi d{\left(\frac{d\theta }{df}\right)}^{-1}.$$

In Figures 3c and 3d, the solid black lines exhibit *θ* expected from a time‐shift due to *v*_*ϕ*_ = *v*_*E*×*B*_, and the magenta lines result from linear fits, with corresponding velocities shown in the legend. For case A, *v*_*ϕ*_ ≈ *v*_*E*×*B*_, while for B, *v*_*ϕ*_ ≈ *v*_*E*×*B*_ + 500 m/s, which is consistent with results using *X*_*corr*_. Additionally, the wrap‐arounds at *f* ≈ 1,750 Hz (Case A) and *f* ≈ 1,250 Hz (Case B) provide direct estimates of the wavelengths (Pfaff et al., 1997). Here, they occur for *λ* = 2*d* and using the frequencies above‐mentioned and *v*_*ϕ*_ from the fits, the estimated interferometric baselines are 1.1 and 0.97 m, which is close to the mNLP separation *d* = 1 m.

Figures 3e and 3f show the PSDs of mNLP fluctuations. Coherent frequencies can be converted to wavenumbers since from panels (c and d), the conversion appears linear. For A, enhanced coherent PSD is seen between *k* ≈ 1 m^−1^ and *k* ≈ 3 m^−1^, and for Case B, enhancement is seen around *k* ≈ [0.5 − 1] m^−1^, that is, close to *λ* = 15 m (vertical dashed line). Analysis suggests the presence of irregular structures reaching *λ* comparable to and shorter than the cold (*T*_*i*_ = 1000 K) oxygen ion gyroradius $\rho_{O^{+}}\approx 2.9$ m.

Figures 3g–3l show similar analysis performed for the *E*‐field using ΔΦ_3_ and ΔΦ_4_ measured on opposite sides of the payload. During the intervals shown, ΔΦ's intersects suggest that **d** was roughly aligned with **v**_**E×B**_. The time intervals are offset by about 0.2 s compared to *N*_*e*_ intervals (rotation of ∼45°) since the instruments were not aligned. As for *N*_*e*_, the relation between *θ* and the frequencies is linear with opposite slope signs for each case, but with origins shifted by *π*. This is consistent with the response expected from an *E*‐field perturbation where *θ* and **k** are related through $\theta \left(\omega \right)=\frac{k\cdot d}{2}+\pi$ (Kintner et al., 1984; LaBelle & Kintner, 1989). Using this equation and *d*/2 = 3.25 m, *v*_*ϕ*_ obtained along the *E*‐field interferometer are consistent with the ones calculated using the mNLPs. Estimated wrap‐arounds at *f* ≈ 1,100 Hz (Case A) *f* ≈ 750 Hz (Case B) confirm the baseline of *λ* = *d*/2 = 3.25 m. Furthermore, for both cases shown, enhanced coherent PSD is seen in the range *k* ≈ [0.2 − 1.5] m^−1^ with maxima close to *k* ≈ 0.5 m^−1^ or *λ* = 15 m.

The CPSDs shown in Figure 3 suggest that the fluctuations have low phase velocity in the plasma. Such irregularities have been termed “spatial irregularities” (e.g., Holmgren & Kintner, 1990; Kintner et al., 1987; Stasiewicz & Khotyaintsev, 2001). To investigate a larger part of the flight, the procedure was automated for the 500–780 s interval. CPSDs were computed every 0.02 s using the same method as for Figure 3. For each CPSD, a linear regression was fitted to *θ* for *f* = [150, 1,000] Hz (mNLP) and *f* = [100, 600] Hz (ΔΦ) (based on Figure 3 and testing). *v*_*ϕ*_ was calculated for *θ*s exhibiting reasonably linear trends, that is, fits with more than 20 variables and an adjusted coefficient of determination *R* ^2^ > 0.7 (mNLP) and *R* ^2^ > 0.9 (ΔΦ). Structures are highly intermittent and these adjustable parameters (length of interval, frequency range, goodness of fit, threshold for fluctuations being “coherent,” etc.), as well as an unknown exact angle between the interferometer baselines and the structures give rise to uncertainties in the velocity calculations: as seen in Figure 3, significant spread in *θ* exist, and automatized fits may not always capture the trend adequately. Further developing robust interferometric methods is however left for future work.

**Figure 4:**
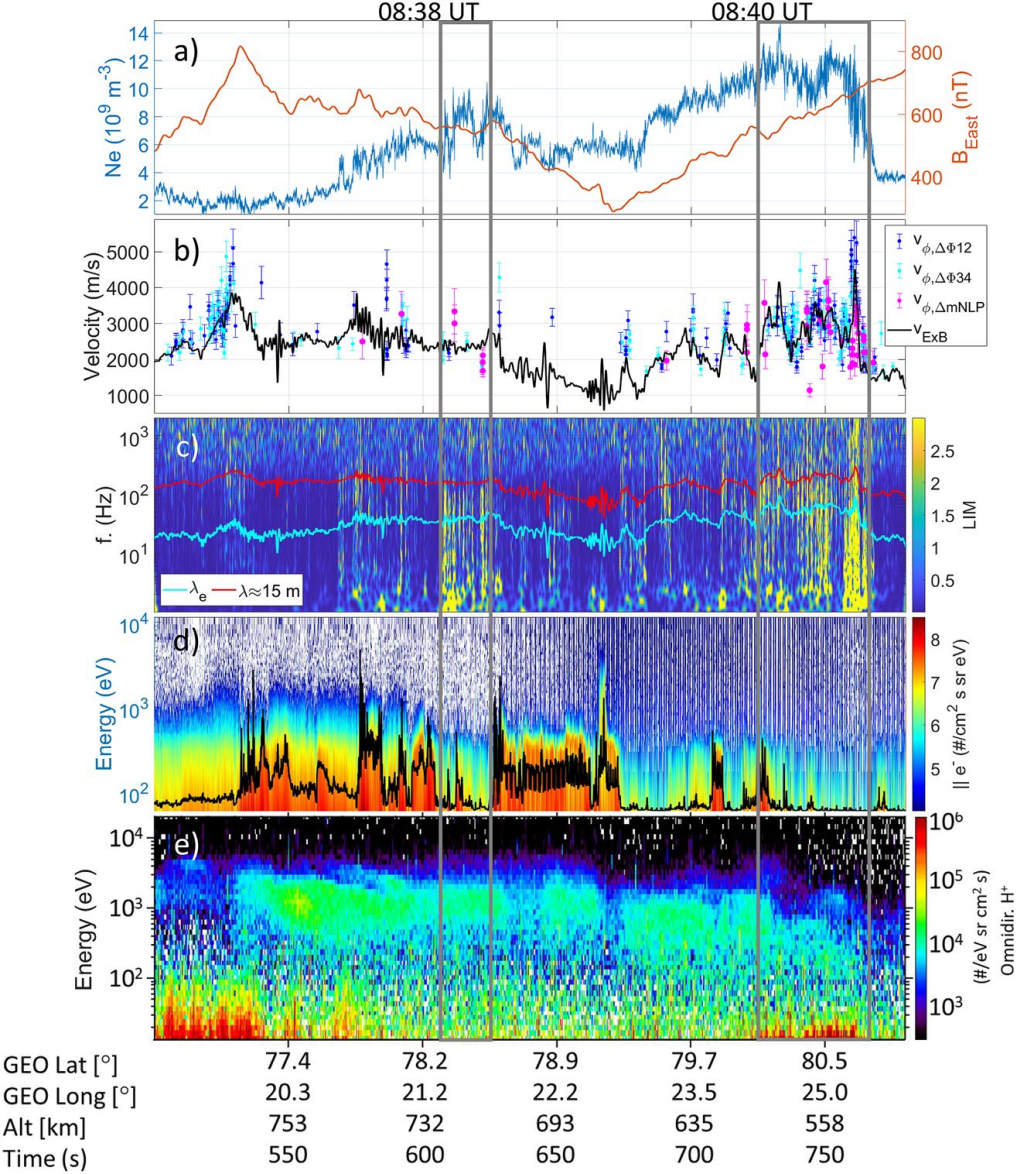
(a) *N*_*e*_ and *B*_*East*_. (b) Velocities obtained using Cross‐spectral density analysis on multi‐needle Langmuir probe (magenta) and *E*‐field (blue/cyan) data for different probe pairs. (c) Local intermittency Analysis of mNLP3 current with the electron inertial length *λ*_*e*_ and *λ* ≈ 15 m superimposed. (d) Electron number flux parallel to **B** (color) and total number flux (black line, a.u.). (e) Omnidirectional ion number flux. Times from Figure 1 are annotated at the top.

Results are presented in Figure 4. Panel (a) shows *N*_*e*_ and the eastward component of the magnetic field (*B*_*East*_). Positive slopes in *B*_*East*_ are expected to correspond to downward currents (Lühr et al., 1996). Figure 4b shows *v*_*ϕ*_ with confidence intervals (±2*σ* on the regression coefficients) obtained from the automated method applied to mNLP (magenta) and different *E*‐field probe pairs (blue/cyan). The black line shows *v*_*E*×*B*_. Calculated *v*_*ϕ*_s are relatively noisy and it is easier to obtain linearity for the *E*‐field probes; however, values appear generally consistent with *v*_*E*×*B*_ suggesting low phase velocities in the plasma. Most of the structures with *θ* exhibiting linear trends for both *N*_*e*_ and *E* are clustered between about 725 and 765 s. Significant *E*‐field and some sparse *N*_*e*_ fluctuations are also observed at other times, for example, at about 505–530 s and around 610 s.

To investigate *N*_*e*_ inhomogeneities, we applied Local Intermittency Analysis (LIM), which accentuates times and frequencies at which the power is intensified compared to the average power in the fluctuations (Farge, 1992; Tam et al., 2005). The LIM was calculated using Morlet wavelets (Torrence & Compo, 1998), and results for mNLP3 are shown in Figure 4c. Several intervals with significant LIM are observed, with the most prominent highlighted with the gray boxes and encompassing where most nondispersive *N*_*e*_ irregularities are detected. For these, frequencies can be translated to wavelengths (Holmgren & Kintner, 1990) and, to guide the eye, the electron inertial length *λ*_*e*_ and the characteristic *λ* ≈ 15 m for SuperDARN backscatter are shown. Several intervals exhibit intermittent structures with *λ* < *λ*_*e*_ and *λ* ≤ 15 m.

Panels (d and e) exhibit the parallel (±10° with respect to **B**) electron and omnidirectional ion number fluxes, respectively. Most electron precipitation has energies below 1 keV, as expected for cusp aurora (e.g., Vontrat‐Reberac et al., 2001). In panel (d), the number flux sum is also shown as a black line for visualization purposes. From Figure 4, it appears that most spatial and nondispersive small‐scale irregularities are detected outside of regions with the strongest precipitation, and within intervals where *B*_*East*_ has roughly positive slope. Also, many of the 15 m *N*_*e*_ irregularities coincide with ion upflows around 720–760 s (pitch angle of about 135°, not shown).

## 5 Summary and Interpretation

This work presents a detailed investigation of *N*_*e*_ and *E*‐field fluctuations in the polar ionosphere. While interpreting in‐situ “single‐point” observations of irregularities and turbulence is generally ambiguous (Fredricks & Coroniti, 1976; Guio & Pécseli, 2021; Temerin, 1978), interferometry techniques separate the spatial and temporal scales of *F* region irregularities in the cusp. The analysis revealed nondispersive irregularities with low phase velocity in the plasma frame, allowing to estimate irregularity power in wavenumber domain, which is essential to interpret observations with respect to turbulence theories (Kintner & Seyler, 1985). Analysis shows the presence of enhanced spatial oscillations around *k* ∼ 0.5 m^−1^, that is, close to the characteristic *λ* = 15 m for SuperDARN backscatter, as well as extending down to $k\rho_{O^{+}}\geq 1$. This provides in‐situ confirmation of decameter‐scale *N*_*e*_ irregularities moving with the **E** × **B**, an essential hypothesis to derive line‐of‐sight velocities from HF radars and large‐scale convection patterns from SuperDARN (e.g., Makarevich & Bristow, 2014; Ruohoniemi et al., 1989, 1987; Villain et al., 1985).

This study supplements previous detection of spatial irregularities, see reviews by LaBelle et al. (1986) and Temerin and Kintner (1989) and references therein. In the upper polar ionosphere, examples include *E*‐field inhomogeneities detected by Kelley and Mozer (1972) and zero‐frequency turbulence commonly identified from “fingerprint” patterns (for *λ* < *d*) (Temerin, 1978, 1979). Also, cusp *E*‐field waves above 5,700 km have been shown to have low *v*_*ϕ*_ (Angelopoulos et al., 2001). For *N*_*e*_, spatial irregularities were frequently observed at higher altitudes using satellites (Holmgren & Kintner, 1990; Kintner et al., 1987; Reiniusson et al., 2006; Stasiewicz & Gustafsson, 2000; Stasiewicz et al., 2000); however, our high‐resolution observations of coinciding *N*_*e*_ and *E*‐field fluctuations for which the phase velocities (and consequently wavenumbers/wavelengths) could be assessed are unique, especially in the cusp *F* region where scintillations and wide HF spectra are also observed.

Decameter‐scale *E*‐field and *N*_*e*_ fluctuations are identified and velocities obtained for both quantities are consistent, suggesting they are locked. Poleward of the aurora (Figure 2), they coincide with larger‐scale (hectometers) steep *N*_*e*_‐variations, suggesting they result as secondary processes (Moen et al., 2012). 10‐m *F* region *N*_*e*_ structures have previously been suggested to spawn down from km‐scale gradients (Moen et al., 2012; Spicher et al., 2014) and, for T2‐L, observations of intermittent fluctuations at specific wavenumber ranges are shown (especially clear for *E*‐field). Drift‐waves (e.g., Pécseli, 2015) and GDI can be excited on density gradients. GDI is often regarded as dominant in the *F *region (Lamarche et al., 2020; Tsunoda, 1988) and can indeed cause decameter‐scale structures directly, provided favorable conditions exist (Makarevich, 2017). While the flow was dominantly perpendicular to the rocket motion, the ∼11 m structures seen in Figure 3f) may result from GDI on the larger‐scale *N_e_*‐variation since *v*_*E*×*B*_ exhibited a southward component (anti‐parallel to the along‐track variation). In fact, Makarevich (2017) showed that, for strong convection in the *F* region, the most favorable conditions for GDI occurred when ∇*N*_*e*_ and *v*_*E*×*B*_ are not exactly aligned. Observations of decameter‐scale structures on *N*_*e*_‐variations with opposite signs when the flow exhibited a northward component (not shown) further support GDI. Moreover, linear kinetic theory also suggests GDI grows at *kρ*_*i*_ > 1 for typical ionospheric conditions for large *v*_*E*×*B*_, but additional theoretical developments are needed (Gary & Cole, 1983), also to assess the *E*‐field oscillations at *k* ≈ 0.5 m^−1^. Note that our cusp observations of intermittent fluctuations are in line with findings from Tam et al. (2005), who showed the presence of highly intermittent *E*‐field fluctuations lasting a few milliseconds in the auroral oval. Tam et al. (2005) suggested this to be indications that broadband extremely low frequency fluctuations were caused by sporadic and localized coherent structures interacting nonlinearly (Chang, 2001).

Not all small‐scale *N*_*e*_ fluctuations were co‐located with larger‐scale *N*_*e*_‐variations (see Figure 3e) and other mechanisms were likely at play during the flight. Particle precipitation is believed to be important at high latitudes and for HF backscatter (e.g., Dyson & Winningham, 1974; Moen et al., 2002; Ponomarenko et al., 2007). Here, the largest occurrence of *N*_*e*_ irregularities was detected poleward of the cusp, adjacent to regions with intense electron fluxes. Precipitation was thus unlikely a dominant direct driver of decameter‐scale structures, but it may have caused “seed” irregularities for secondary processes (Moen et al., 2012; Oksavik et al., 2012).

As seen in Figure 2c, a flow shear was observed at about 762 s. The shear scale‐size matches that used in numerical simulations showing that KHI could quickly cause irregularities at scintillation scales in the cusp (Spicher et al., 2020). However, KHI is mostly a long‐wavelength, low‐frequency, instability and therefore it fails to explain direct creation of $k\rho_{O^{+}}\geq 1$ structures (Ganguli et al., 1994). Secondary processes such as the inhomogeneous energy density driven instability (Ganguli et al., 1985) could operate; however, the shear frequency (∼1 Hz) is low compared to the *O* ^+^ gyrofrequency (∼40 Hz), suggesting that this instability is not dominant (Ganguli et al., 1994).

Alternatively, the coincidence between positive slopes in *B*_*East*_, ion upflows and the largest concentration of spatial structures is intriguing. Upper *F* region *N*_*e*_ enhancements and the presence of larger‐scale fluctuations in the return current (as seen poleward of the cusp in Figure 4) are consistent with simulations and observations associated with the ionospheric Alfvén resonator (Cohen et al., 2013; Streltsov & Lotko, 2008). Especially, dispersive Alfvén waves (Stasiewicz et al., 2000) and electrostatic slow ion cyclotron/acoustic waves (for hot ions) have been suggested as processes with low phase velocity and broadband low‐frequency *E*‐field fluctuations (Seyler & Wahlund, 1996; Seyler et al., 1998; Wahlund et al., 1998). Altogether, this study provides new insights into the behavior of small‐scales *N*_*e*_ and *E*‐field irregularities as well as physical context for what causes wide SuperDARN spectra in the cusp, and further analysis will hopefully help resolve the exact mechanism(s) taking place.

## Data Availability

Data from the TRICE‐2 missions can be found at: https://phi.physics.uiowa.edu/science/tau/data0/rocket/SCIENCE/TRICEII_Mission/. mNLP data is available at https://archive.sigma2.no/pages/public/datasetDetail.jsf?id=10.11582/2022.00032 (Clausen, 2022). Super Dual Auroral Radar Network data is available at https://www.frdr-dfdr.ca/repo/collection/superdarn with basic data analysis software at https://zenodo.org/record/4435297. The imager data are available at http://tid.uio.no/plasma/aurora/. The wavelet analysis is based on an adapted version of a (Morlet) wavelet software provided by C. Torrence and G. Compo (Torrence & Compo, 1998, latest version is available at URL: http://atoc.colorado.edu/research/wavelets/). The panel showing ion data was obtained using SPEDAS V3.2 (Angelopoulos et al., 2019).
